# Structure-based prediction and identification of 4-epimerization activity of phosphate sugars in class II aldolases

**DOI:** 10.1038/s41598-017-02211-3

**Published:** 2017-05-16

**Authors:** Seon-Hwa Lee, Seung-Hye Hong, Jung-Ung An, Kyoung-Rok Kim, Dong-Eun Kim, Lin-Woo Kang, Deok-Kun Oh

**Affiliations:** 1 Department of Bioscience and Biotechnology, Konkuk University, Seoul, 05029 Republic of Korea; 2 Department of Biological Sciences, Konkuk University, Seoul, 05029 Republic of Korea

## Abstract

Sugar 4-epimerization reactions are important for the production of rare sugars and their derivatives, which have various potential industrial applications. For example, the production of tagatose, a functional sweetener, from fructose by sugar 4-epimerization is currently constrained because a fructose 4-epimerase does not exist in nature. We found that class II d-fructose-1,6-bisphosphate aldolase (FbaA) catalyzed the 4-epimerization of d-fructose-6-phosphate (F6P) to d-tagatose-6-phosphate (T6P) based on the prediction via structural comparisons with epimerase and molecular docking and the identification of the condensed products of C3 sugars. *In vivo*, the 4-epimerization activity of FbaA is normally repressed. This can be explained by our results showing the catalytic efficiency of d-fructose-6-phosphate kinase for F6P phosphorylation was significantly higher than that of FbaA for F6P epimerization. Here, we identified the epimerization reactions and the responsible catalytic residues through observation of the reactions of FbaA and l-rhamnulose-1-phosphate aldolases (RhaD) variants with substituted catalytic residues using different substrates. Moreover, we obtained detailed potential epimerization reaction mechanism of FbaA and a general epimerization mechanism of the class II aldolases l-fuculose-1-phosphate aldolase, RhaD, and FbaA. Thus, class II aldolases can be used as 4-epimerases for the stereo-selective synthesis of valuable carbohydrates.

## Introduction

Rare sugars can be used as starting materials for the synthesis of potential natural products with important biological activities for applications in the food, pharmaceutical, medicinal, and chemical industries^1, 2^. Sugar 4-epimerization is a valuable reaction for the production of rare sugars and their derivatives. For economic reasons, researchers have tried to find a fructose 4-epimerase converting fructose to tagatose, which is a functional sweetener^3^, but without success. Moreover, because there are only four sugar 4-epimerases available, including ribulose 5-phosphate 4-epimerase (AraD), UDP-*N*-acetylglucosamine 4-epimerase, UDP-galactose 4-epimerase, and tagaturonate-fructuronate 4-epimerase^4, 5^, the discovery of a new 4-epimerase is of industrial importance.

Aldolases are widely used in the chemical and pharmaceutical industries^6–8^ because they function in diverse catabolic and anabolic processes and produce various stereoisomers. Enzymatic synthesis using aldolases for C-C bonding has advantages over chemical synthesis because highly stereo-selective products can be obtained under mild conditions^8, 9^. Although class II aldolases and sugar 4-epimerases have different enzyme classification (EC) numbers as lyase (EC 4) and isomerase (EC 5), respectively, their mechanisms share a common enediolate intermediate^10–12^. l-AraD was proposed to be an aldolase mimic^10^ and d-tagatose-1,6-bisphosphate aldolase (AgaY) has a tagatose-1,6-bisphosphate epimerase activity^13, 14^. These results suggest that class II aldolases can be used as sugar 4-epimerases.

Bioinformatic tools can be used to predict the evolutionary relationships, functions, and substrate preference of enzymes based on their structures and reaction mechanisms. Studies on the docking of enzymes with substrates or analog libraries have identified novel enzyme functions^15, 16^. To predict the specific reaction more precisely, a comparative analysis of the mechanisms for substrate selection should be merged with the docking studies. In the present study, we discovered the unreported 4-epimerization activity and catalytic residues of d-fructose-1,6-bisphosphate aldolase (FbaA) for d-fructose-6-phosphate (F6P) by finding a suitable substrate based on structure- and mechanism-based prediction. Moreover, we extended this discovery to class II aldolases.

## Results and Discussion

### Structural comparison of aldolases and epimerases

**Figure 1:**
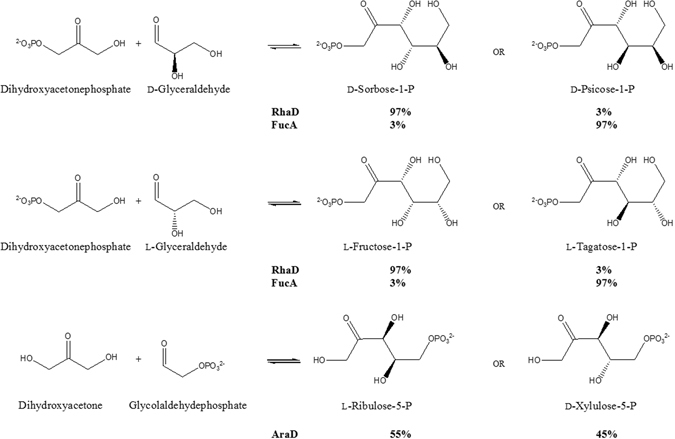
4-Epimerization reactions of sugar phosphates by class II aldolases and epimerase. The products of the enzymatic reactions of class II aldolases and epimerase are 4-OH epimer forms. Class II aldolases can catalyze the 4-epimerization for the specific substrates.

AraD catalyzed the 4-epimerization reaction through the aldol cleavage-condensation mechanism of class II aldolases as a result of its flexible stereo-selectivity, suggesting that class II aldolases have a potential 4-epimerization activity^13^. To investigate the sugar 4-epimerase activities for these enzymes, we sorted 22 sugar-related epimerases and aldolases from the Structural Classification of Proteins-extended (SCOPe) database. We selected one 4-epimerase, AraD, and four class II aldolases, l-fuculose-1-phosphate aldolase (FucA), RhaD, FbaA, and AgaY, based on the commonalities of their substrates as phosphate sugars (Table S1). AraD catalyzed the 4-epimerization reaction of l-ribulose-5-phosphate (l-R5P) to d-xylulose-5-phosphate (X5P)^13^. FucA and RhaD synthesized not only l-tagatose-1-phosphate (l-T1P) and l-fructose-1-phosphate (l-F1P) from l-glyceraldehyde (l-GA)^17^, but also d-psicose-1-phosphate (P1P) and d-sorbose-1-phosphate (S1P) from GA (Fig. 1)^2^. The amino acid sequence similarity of AraD was 24%, 13%, 10%, and 16% to FucA, RhaD, FbaA, and AgaY, respectively, whereas the structural similarity calculated using the Flexible structure AlignmenT by Chaining Aligned fragment pairs allowing Twists (FATCAT) rigid algorithm^18^ was 92%, 92%, 63%, and 61%, respectively. The tetrahedral arrangement by three histidine residues coordinating a Zn^2+^ and a catalytic acidic residue (Glu or Asp) in the active site of each enzymes was spatially conserved (Figs S1 and S2). Despite low sequence similarity, the results of structural conservation of active sites suggested a strong connection between these enzymes.

### Identification of 4-epimerization activity of FbaA

**Figure 2:**
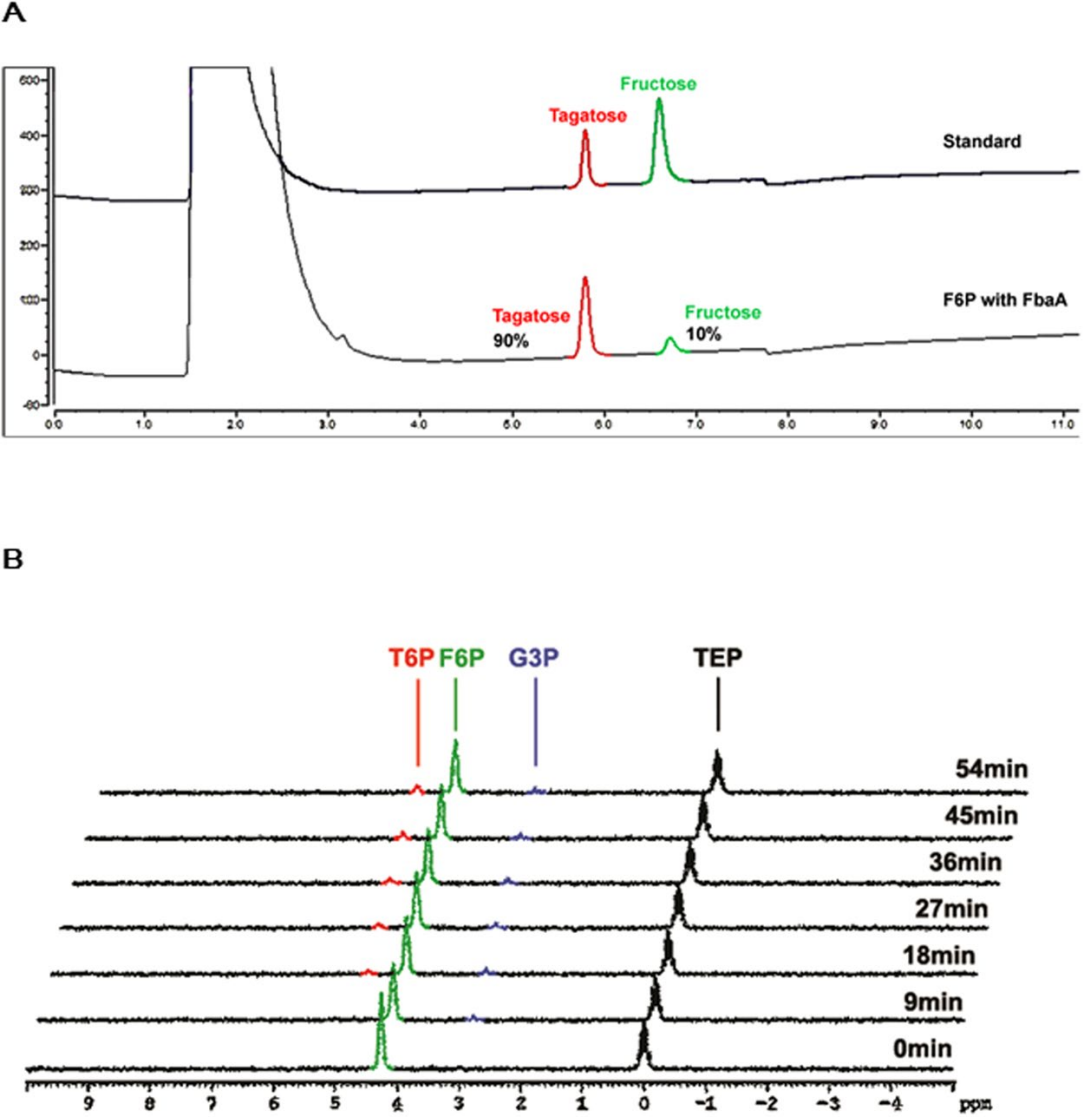
Bio-LC diagram and ^31^P-NMR spectra for the reaction of F6P with FbaA. (**A**) Bio-LC diagrma of T6P biocoversion from F6P by FbaA. Reactions were carried out at 50 ^o^C for 3 h in 50 mM Tris-HCl buffer containing 0.5 mM F6P with FbaA. (**B**) ^31^P-NMR spectra for the reaction of F6P with FbaA. TEP was ued as the internal standard.

In general, aldolases are highly specific dihydroxyacetone phosphate (DHAP)-dependent enzymes. However, AraD can use dihydroxyacetone (DHA) as the donor substrate^10^. Therefore, we extended the use of DHA as the donor for the condensation reaction of FbaA with d-glyceraldehyde-3-phosphate (G3P) and GA as the acceptors. As a result, F6P and T6P were synthesized from the condensation of G3P and DHA, while d-fructose-1,6-bisphosphate (FBP) or d-fructose-1-phosphate (F1P) was synthesized from DHAP and G3P or GA, respectively (Table S2), but the epimer products d-tagatose-1,6-bisphosphate (TBP), T1P, and tagatose were not observed. The conversion of F6P to T6P by FbaA was confirmed by Bio-LC and ^31^P-nuclear magnetic resonance (NMR) spectroscopy (Fig. 2). Although *Escherichia coli* FbaA did not epimerize FBP to TBP due to having a small cavity volume, it can epimerize the smaller substrate F6P to T6P^19^.

When the kinetic values of F6P and T6P were measured (Table S3), the *k*_cat_/*K*_m_ of FbaA was approximately 9-fold higher for F6P than for T6P according to the measurement of kinetic parameters; hence, the specificity of F6P to the active site of FbaA is 9 times more favorable than T6P. The equilibrium ratio established between F6P and T6P by FbaA was 10:90 (Table S4). However, T6P is not found during glycolysis. Thus, the question of why a 4-epimerization activity of FbaA has not been reported remained. To answer this question, the enzymatic conversions for F6P were investigated. The *k*_cat_/*K*_m_ of d-fructose-6-phosphate kinase (PfkA) for F6P phosphorylation is 1,700-fold higher than that of FbaA for F6P epimerization^20^, suggesting that F6P is a more preferred substrate for PfkA than FbaA. The distribution of F6P, T6P, and FBP was determined by varying the ratio of PfkA to FbaA (Table S5). T6P was produced from F6P by FbaA without PfkA, but its production was markedly reduced by adding PfkA and was abolished when PfkA and FbaA were present in equal amounts. Thus, the 4-epimerization activity of FbaA is normally repressed by PfkA *in vivo*. To eliminate the inhibitory effect of PfkA, we designed an *in vitro* reaction. A three-enzyme cascade reaction, involving FbaA, fructose kinase (ScrK), and phosphatase, was constructed. In the reaction, 50 mM fructose was converted to 45 mM tagatose for 16 h (Fig. S3). This conversion (90%) of a sugar to tagatose was the highest ever reported and was higher than that (approximately 44% at 60 °C) of galactose to tagatose by l-arabinose isomerase^21^.

### Mechanism of FbaA for F6P 4-epimerization

**Figure 3:**
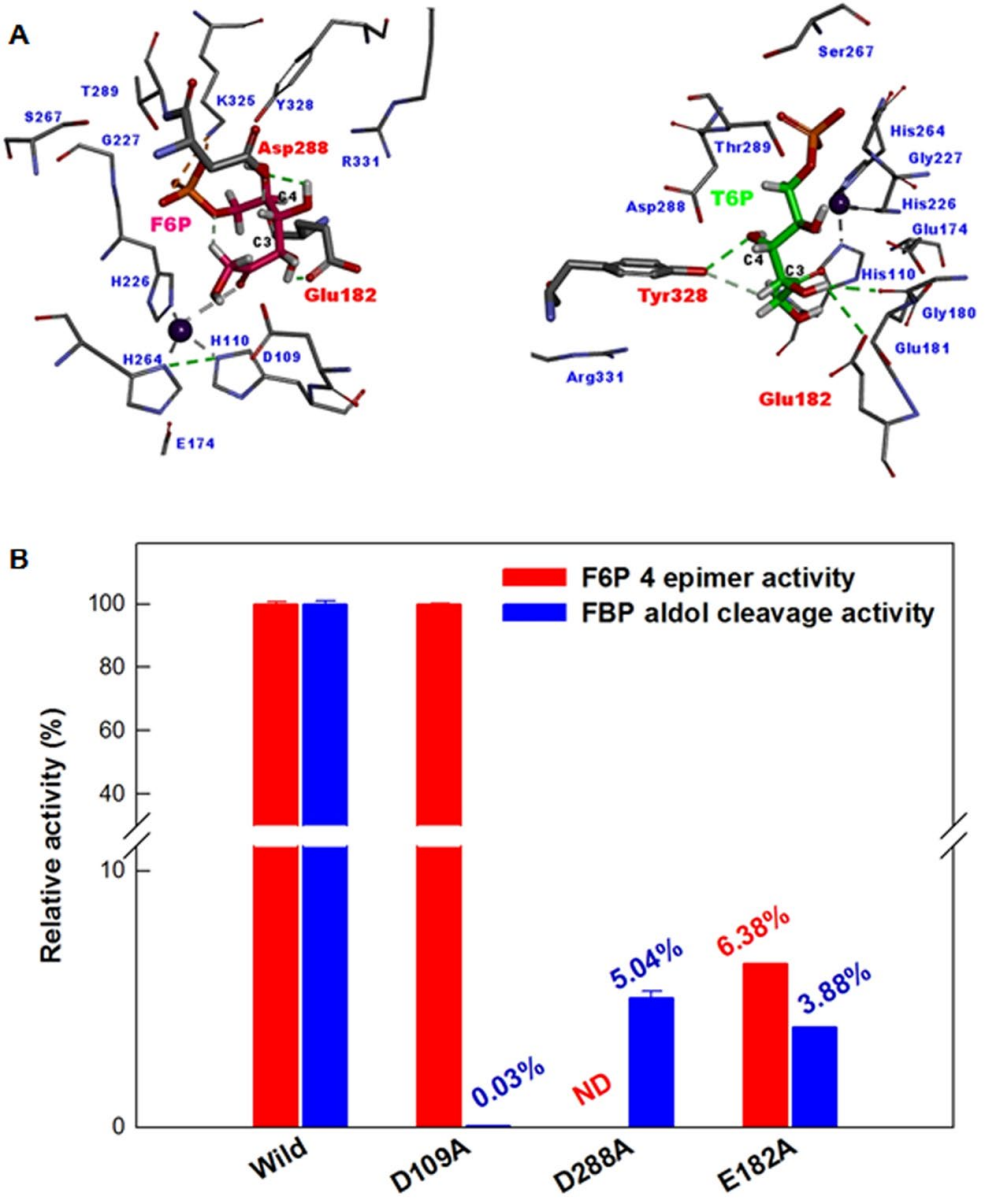
(**A**) Docking model of FbaA with F6P, T6P, and catalytic residues. They represent pink, green, and red colors, respectively. Green dashed lines represent interactions between the substrates and catalytic residues. Grey dashed lines represent interactions with Zn^2+^. (**B**) Aldol cleavage and 4-epimerization activities of the wild-type and catalytic residue variant FbaAs from *E. coli*. Asp288 mediate aldol cleavage via iraction with FBP, whereas Glu182 and Asp288 mediate 4-epimerization via interaction with F6P. Data represent the means of three experiments and error bars represent standard dviations.

After we had found the 4-epimerization activity of FbaA, the investigation of the catalytic residues and mechanism for the 4-epimerization activity became meaningful. To determine each catalytic residue of FbaA for 4-epimerization, the possible products, including F1P, F6P, T6P, FBP, and d-fructose, were docked to FbaA. 4-Epimerization activity could be considered as forming hydrogen bonds with C3- and C4-OH for electron transfer. The docking of F6P or T6P to FbaA revealed hydrogen bonds between Glu182 and C3-OH and between Asp288 and C4-OH; or between Glu182 and C3-OH and between Tyr328 and C4-OH, respectively (Fig. 3A). These hydrogen bonds suggest the possibility of F6P and T6P 4-epimerization. The docking of FBP or F1P to FbaA revealed hydrogen bond formation between Asp109 and C3-OH. However, d-fructose did not form hydrogen bonds with FbaA (Fig. S4).

To identify the related residues for catalysis, we evaluated F6P 4-epimerization and FBP aldol cleavage activities using FbaA variants (Fig. 3B and Table 1). Asp 109 is known as a catalytic residue of FbaA for FBP aldol-cleavage reaction. D109A for FBP showed no activity, but its activity for F6P 4-epimerization was similar to that of the wild-type FbaA. Asp288 and Glu182 were candidates for the predicted catalytic residues for 4-epimerization of F6P and T6P by the molecular docking models and mutation results (Fig. 3). The epimerization activities of D288A for F6P and Y328A for T6P were not detected (Fig. S5). The epimerization activity of E182A for F6P was only 6% of the wild-type enzyme activity. Thus, Asp109, E182, Asp288, and Ty328 critically affected the enzyme activity.

To determine the relationship between the structure and the catalytic activity of FbaA, the secondary structures of the wild-type and variant enzymes were analyzed using circular dichroism (CD) spectroscopy in the far-UV and near-UV spectral regions. The CD spectra of the D109A, E182A, D288A, and Y328A variants showed high similarity with those of the wild-type enzyme (Fig. S6). They showed characteristic CD spectra with a negative band at 222 nm revealing a high content of alpha-helix structure. These results indicate that the point mutation of those sites did not result in a conformational change of the variant enzymes.

These molecular docking models and mutation results indicate that FbaA is a dual-activity enzyme that catalyzes two reactions, FBP aldol cleavage and F6P 4-epimerization, using other catalytic residues within the same binding pocket. When F6P and FBP are bound to the active site of FbaA (Fig. S7A), the different orientations can be explained by the enzyme having different catalytic residues for aldol cleavage and 4-epimerization, and two different G3P poses that correspond to each position of F6P and T6P in the models (Fig. S7C).

For further analyses, we determined the residues for the coordination of catalytic Zn^2+^. The refined structure of FbaA showed that His110 and His264 were bound to Zn^2+^ by rotating their buried imidazole rings to the catalytic site, while His226, which formed a hydrogen bond with Glu175, and a water molecule were bound to the same Zn^2+^, thereby enhancing the stability of the catalytic residue^22^. The 4-epimerization activity was abolished in the triple variant H110A-E175A-H264A (Table 1). The *k*_cat_/*K*_m_ of the H226A variant for F6P 4-epimerization was comparable with that of the wild-type enzyme. Thus, Zn^2+^ might bind to His110, His264, and Glu175; and His226 is not critical for 4-epimerization.

**Figure 4:**
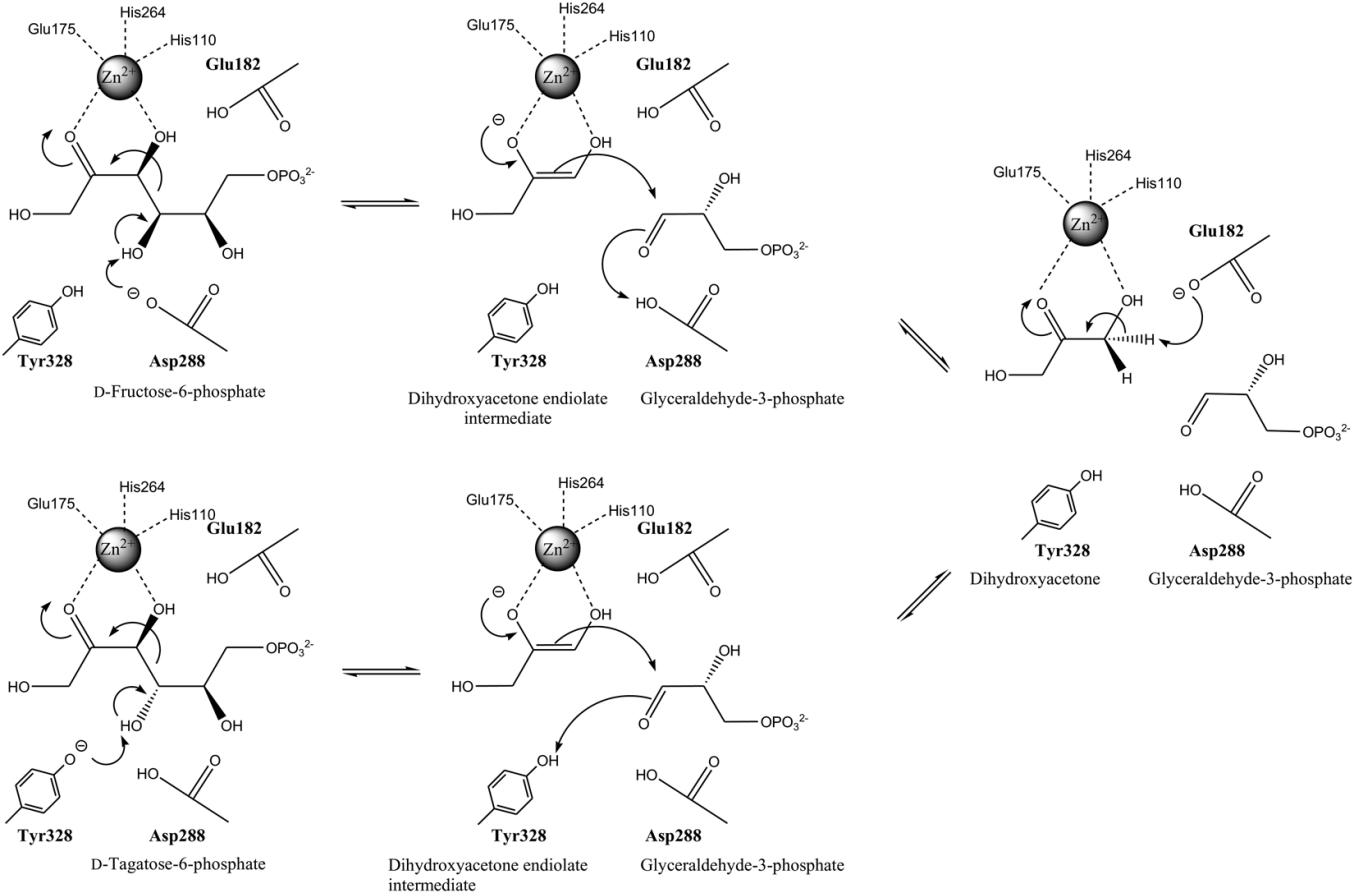
Proposed mechanism of FbaA for reversible 4-epimerization. Asp288 or Tyr328 deprotonates the C4-OH of F6P or T6P, splitting F6P or T6P into the enediolate of DHA and G3P. The neutral or acidic form of Glu182 on the opposite face protonates or deprotonates the C3 in the enediolate of DHA or DHA, respectively.

Based on our findings, we propose a mechanism for F6P 4-epimerization by FbaA based on the catalytic mechanism of Asp109 for FBP^12^. The D288A and Y328A variants could not cleave the C-C bond of F6P and T6P, respectively (Fig. S5). Therefore, 4-epimerization requires the acidic form of the catalytic residue Asp288 or Tyr328 which deprotonates the C4-OH of F6P or T6P, respectively. The phosphate sugar promotes C-C bond cleavage to form the enediolate of DHA and G3P with Zn^2+^ bound to His110, His264, and Glu175 (Fig. 4). The neutral form of Glu182 on the opposite face protonates the newly formed C3 in the enediolate of DHA, resulting in the formation of DHA and G3P as intermediates. The rotation of the aldehyde group in G3P causes a shift from the original position of F6P or T6P to that of T6P or F6P for 4-epimerization, respectively (Fig. S7). The acidic form of Glu182 deprotonates the C3 in DHA to form the enediolate of DHA, which is then condensed with G3P to form T6P or F6P, respectively. This mechanism differs from that of aldol cleavage, which requires the catalytic residue Asp109; its acidic form deprotonates the C4-OH of FBP, which is cleaved into DHAP and G3P with Zn^2+^ bound to His110, His264, and His226^22^.

### 4-Epimerization reactions by other class II aldolases

We proposed two catalytic residues for the 4- epimerization activity of FbaA. These residues are different from the well-known catalytic residues of aldolase activity. The different catalytic residues for epimerization suggest that aldolases can be used as epimerases by finding the relevant catalytic residues. Thus, the catalytic residues for epimerization of other class II aldolases have been investigated. AraD and FucA have similar structures and mechanisms. The catalytic residue for epimerization between l-R5P and X5P in AraD was predicted as Asp76 by comparison with the structure of FucA, but the actual catalytic residues later identified as Asp120 and Tyr229^13^. Although direct evidence has not been presented yet, the catalytic residue of FucA for the epimerization between l-F1P and l-T1P has been suggested as Tyr209^17^, which is different to that of FucA for aldol cleavage (Glu73).

As revealed in this study, the catalytic residues of FbaA for the epimerization of F6P and T6P are Asp288 and Tyr328, respectively, and Asp109 is not involved. The catalytic residue of the class II aldolase RhaD for aldol cleavage is known as Glu117, whereas those for epimerization between P1P and S1P are unknown^2^. The pattern of P1P and S1P produced from DHAP and GA of E117A in Bio-LC was similar to that of the wild-type enzyme (Fig. S8). Thus, Glu117 is not involved in epimerization. In a ligand-docking study, Glu200 formed hydrogen bonds with C4-OH of S1P, and Thr115 was near to C4-OH of P1P (Fig. S9). T115A and E200A showed significant reduction in the condensation of P1P and S1P from DHAP and GA, respectively (Fig. S8). These results suggest that these residues are newly identified catalytic residues for 4-epimerization and the stereo-selectivity of aldolases is tunable by the mutation of catalytic residues involved in epimerization.

**Figure 5:**
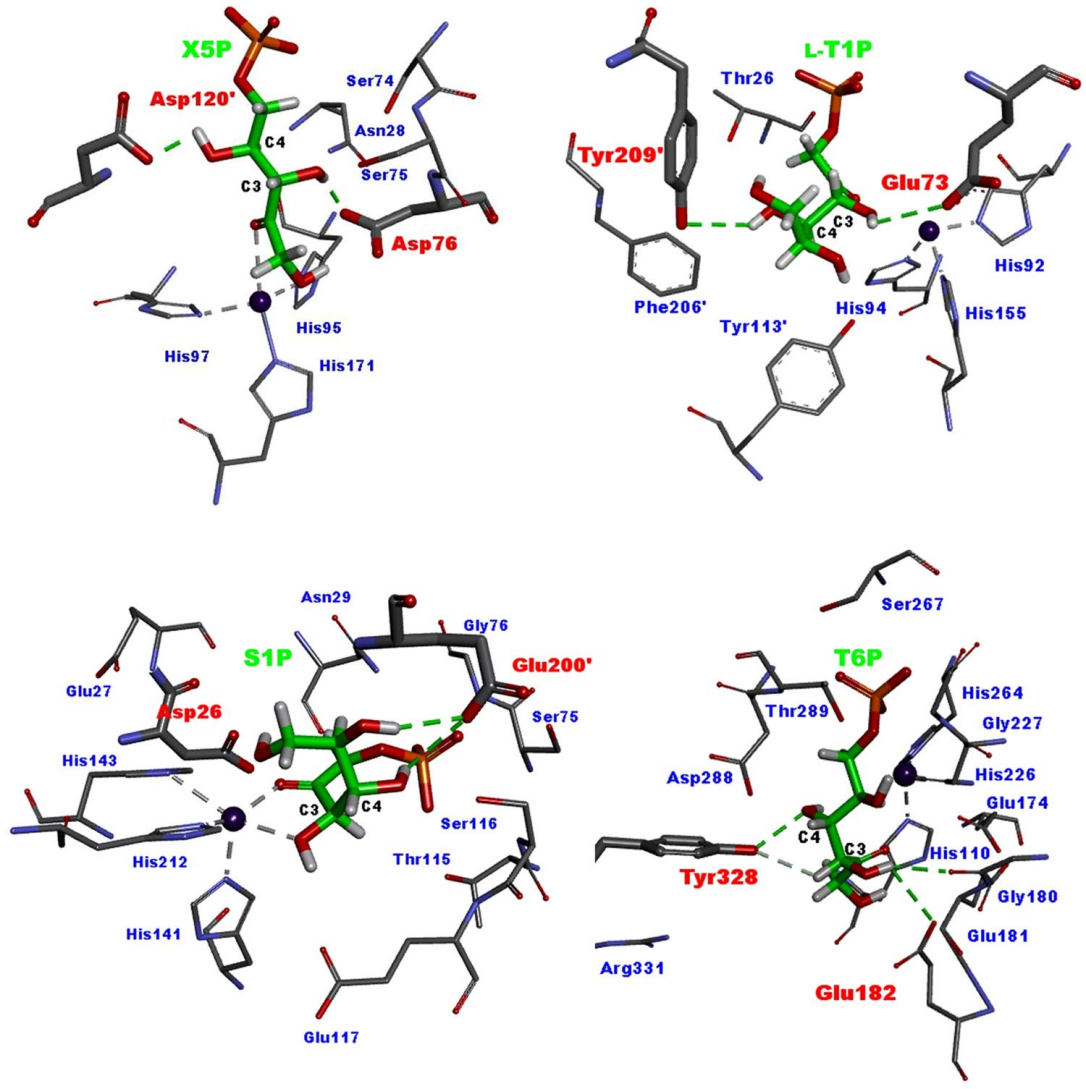
Docking models of AraD, FucA, RhaD, and FbaA for 4-epimerization. The models show the formation of hydrogen bonds at a catalytic acid and a base (bold) when docked with the dominant product of 4-epimerization. One residue forms a hydrogen bond with the C4-OH of the ligand while another forms a hydrogen bond with the C3-OH of the ligand (green), which is responsible for ligand deprotonation, respectively. (**A**) Docking of X5P in the active site of AraD formed by two adjacent subunits. Asp120 and Asp76 are a catalytic acid and a base for 4-epimerization of l-R5P to X5P, respectively. (**B**) Docking of l-T1P in the active site of FucA formed by two adjacent subunits. Tyr209 and Glu73 are a catalytic acid and a base residue for 4-epimerization of l-F1P to l-T1P. (**C**) Docking of S1P in the active site of RhaD formed by two adjacent subunits. Thr200’ and Asp26 are a catalytic acid and a base for 4-epimerization of P1P to S1P, respectively. (**D**) Docking of T6P in the active site of FbaA. Tyr328 and Glu182 are a catalytic acid and a base for 4-epimerization of F6P to T6P, respectively.

The epimerization reaction mechanisms of the class II aldolases FucA, RhaD, and FbaA can be elucidated as follows: When suitable substrates for these aldolases are selected, the enzymes catalyze 4-epimerization reactions using catalytic residues that are distinct from those used for aldol cleavage reactions within the same pocket (Fig. 5). Each substrate is split into two cleavage intermediates with one repositioned for 4-epimerization, and they are condensed into the epimer product. The 4-epimerization reactions of class II aldolases were developed using different substrates to those of aldol cleavage reactions based on identification of suitable epimers through molecular docking. This method could be used to synthesize tailor-made natural and unnatural carbohydrates with controlled stereo-selectivity^8, 14^. These results may help about the understanding of the frequent promiscuity of enzymes in nature. Our structural analysis and substrate binding data for class II aldolases will help in future studies to find other catalytic activities of enzymes based on the analysis of their conserved structural regions.

## Methods

### Database search and secondary structure comparison

Structures were identified and classified using Protein Data Bank (PDB) and SCOPe^23^. Information on reaction mechanisms was obtained from Mechanism, Annotation and Classification in Enzymes (MACiE)^24^. The similarity between single chain structures in each protein was calculated in PDB using the default flexible structure alignment by the FATCAT rigid algorithm. Amino acid sequence alignment was carried out with the Clustal W2 multiple pairwise alignment tool using default parameters and a Gonnet 250 protein weight matrix^25^.

### Cloning and site-directed mutagenesis

Molecular cloning and site-direct mutagenesis were carried out according to standard procedures^26^. The *fbaA* (GenBank accession no. NP_417400.1) and *rhaD* (NP_418338.1) genes of *E. coli* K-12 were amplified by PCR using genomic DNA isolated from *E. coli* K-12 as a template. The forward and reverse primers of *fbaA* contained *Sal*I and *Not*I restriction sites and those of *rhaD* contained *Pst*I and *Not*I. DNA fragments obtained by PCR amplification with *Taq* polymerase (Bioneer, Alameda, CA, USA) were ligated into the pRSF-Duet-1 vector (Novagen, Madison, WI, USA), which was transformed into *E. coli* ER2566 cells and plated on Luria-Bertani (LB) agar containing 0.1 mM kanamycin. A kanamycin-resistant colony was selected and the plasmid DNA was sequenced using a DNA analyzer (ABI Prism 3730xl; Perkin-Elmer, Waltham, MA, USA). Site-directed mutagenesis was carried out using a Quick Change kit (Stratagene, La Jolla, CA, USA) according to the manufacturer’s protocol.

### Heterologous expression and enzyme purification

Recombinant *E. coli* was cultured in a 2-l flask containing 500 ml LB medium and 0.1 mM kanamycin at 37 °C and 200 rpm. When the optical density of the culture at 600 nm reached 0.6, 0.1 mM IPTG was added to induce FbaA and RhaD expression. The culture was further incubated at 16 °C and 150 rpm for 16 h. Recombinant cells were harvested by centrifugation at 2,000 × *g* for 30 min at 4 °C and resuspended in 50 mM phosphate buffer (pH 8.0) containing 300 mM NaCl, 10 mM imidazole, and 0.1 mM phenylmethylsulfonyl fluoride as a protease inhibitor. The cells were disrupted by sonication on an ice bath. Cell debris was removed by centrifugation at 16,000 × *g* for 20 min at 4 °C, and the supernatant was passed through a 0.45-μm filter. The filtrate was applied to an immobilized metal ion affinity chromatography cartridge (Bio-Rad, Hercules, CA, USA) equilibrated with 50 mM Tris-HCl buffer (pH 8.5) containing 300 mM KCl. After extensive washing with the same buffer (pH 8.0) containing 300 mM KCl and 30 mM imidazole, the bound protein was eluted with the same buffer (pH 8.0) containing 300 mM KCl and 300 mM imidazole at a flow rate of 1 ml/min. Active fractions were collected and dialyzed in 50 mM Tris-HCl buffer (pH 8.5 for FbaA and pH 7.5 for RhaD), and the resultant solution was used as the purified enzyme.

### Enzyme activity

The reactions for F6P and T6P 4-epimerization were performed at 50 °C in 50 mM Tris-HCl buffer (pH 8.5) and the condensation reactions of G3P, DHAP, GA, and DHA were performed at 50 °C in 50 mM Tris-HCl buffer (pH 7.0). FBP, F1P, F6P, and T6P were analyzed using a Bio-LC system (ICS-3000; Dionex, Sunnyvale, CA, USA) equipped with an electrochemical detector and Carbo Pac PA1 column, which was eluted at 30 °C at a flow rate of 1 ml/min with mixtures of water, 200 mM NaOH, and 1 M sodium acetate in the following ratios: 35:45:20 (v/v/v) for 0–10 min, 35:15:50 (v/v/v) for 10–15 min, and 35:45:20 (v/v/v) for 15–25 min. F1P and T6P were eluted at 30 °C at a flow rate of 1 ml/min with the mixtures of distilled water, 200 mM NaOH, and 1 M sodium acetate in the following ratios: 30:50:20 (v/v/v) for 0–20 min, 10:20:50 (v/v/v) for 20–30 min, and 30:50:20 (v/v/v) for 30–45 min. The aldol-cleavage activity of FbaA was analyzed through a spectrophotometric coupled enzymatic assay using FBP as substrate. F1P and T6P were detected based on their retention times of 16.9 and 17.7 min. The reactions were performed at 30 °C in 50 mM Tris-HCl buffer (pH 8.5) containing 100 mM potassium acetate, 0.3 mM NADH, coupling enzymes of 5 U/ml glycerophosphate dehydrogenase and 0.5 U/ml triphosphate isomerase, and 0.005 U/ml FbaA by varying the concentration of FBP from 0.05 to 2 mM^27^. A spectrophotometer was used to record the absorbance at 340 nm. S1P and P1P were condensed from DHAP and GA using RhaD and the products were dephosphorylated with phosphatase and analyzed using the same Bio-LC system, which was eluted at 30 °C at a flow rate of 1 ml/min with mixtures of water and 200 mM NaOH in the following ratios: 92:8 (v/v) for 0–30 min, 50:50 (v/v) for 30–40 min, and 92:8 (v/v) for 40–45 min. Sorbose and psicose were detected at 16.7 and 19.5 min. The competitive enzymatic reactions between PfkA and FbaA were carried out in 50 mM Tris-HCl (pH 8.5) containing 10 μM F6P, 0–30 U/ml PfkA, and 0–30 U/ml FbaA for 10 min at 65 °C. One unit (U) of FbaA 4-epimerization activity was defined as the amount of enzyme required to epimerize1 µmol T6P per min at 50 °C and pH 8.5 from F6P. One unit (U) of PfkA was defined as the amount required converting 1 μmol F6P and ATP to FBP and ADP per minute at 65 °C and pH 8.5.

### Determination of kinetic parameters

F6P and FBP concentrations in the range of 0.05–10 mM were used to determine the kinetic parameters for F6P 4-epimerization and FBP condensation. The reactions were performed in 50 mM Tris-HCl buffer (pH 8.5) containing purified FbaA at 50 °C for 10 min and were terminated by adding 200 mM HCl. The kinetic parameters *K*_m_ (mM) and *k*_cat_ (min^−1^) were determined from a Hanes–Wolf plot derived from the Michaelis–Menten equation. To calculate the catalytic constant *k*_cat_, the amount of protein was divided by total molecular mass.

### Determination of FbaA 4-epimerization activity by ^31^P- NMR spectroscopy

^31^P-NMR analysis was carried out at 25 °C and a frequency of 202.46 MHz on an Avance 500 MHz spectrometer (Bruker, Billerica, MA, USA) with a 0.5 s repetition time (0.5 s acquisition/0.0 s relaxations for FbaA). The 4-epimerization activity of purified FbaA was tested using F6P as substrate. The reaction was carried out in 50 mM Tris-HCl (pH 8.5) at a final volume of 1 ml containing 10 µmol TEP, 450 µl D_2_O, 0.4 U purified FbaA protein, and 10 mM F6P in a 5-mm NMR tube. FbaA in the blank used to tune the spectrometer was excluded from the solution. After measuring the blank, FbaA was added and the tube was sealed with a rubber cap, inverted several times, and reinserted into the spectrometer. Data acquisition was initiated when a stable lock signal was obtained. Spectra were recorded at 9-min intervals over 1 h. Chemical shifts were measured relative to the TEP internal standard.

#### *In silico* docking and molecular modeling

Each phosphate sugar (FBP, F6P, F1P, and T6P) and fructose was docked in the active-site pocket of the crystal structure of *E. coli* K-12 FbaA with phosphoglycolohydroxamic acid from the PDB (entry 1B57) using the CDOCKER module of Discovery Studio (DS) 4.0 (Accelrys, San Diego, CA), which was allowed by the license acquired for Professor Deok-Kun Oh. Substrate poses were refined by full-potential final minimization and candidate poses were created using random rigid-body rotations, followed by simulated annealing. The structure of enzyme-ligand complexes was subjected to energy minimization using the CHARMM force field in DS 4.5. The substrate orientation with the lowest interaction energy was selected for subsequent rounds of docking. Candidate poses were created based on random rigid-body rotations followed by simulated annealing. The structures of the protein, cofactor, and their complexes were subjected to energy minimization using the CHARMM force field in DS 4.5. Full-potential final minimization was used to refine substrate poses. The energy-docked conformation of the substrate was retrieved for post-docking analysis using the C-DOCKER module. The substrate orientation with the lowest interaction energy was selected for subsequent rounds of docking. To estimate the binding energy between receptor and ligand, changes in the binding energy (*∆E* _binding_) after docking was defined as *E*_complex_−*E*_ligand_−*E*_receptor_. Structural comparisons with other class II aldolase enzymes as RhaD, FucA, AraD, and AgaY were carried out using the same methodology.

#### CD analysis of the wild-type and variant enzymes

CD spectra of the wild-type and variant enzymes were measured at 190–300 nm with a J-810 spectropolarimeter (Jasco, Dunmow, UK) and a scan rate of 100 nm/min at 20 °C, and data were collected at 0.1 nm intervals using 0.1 cm quartz cells. The protein solutions of the wild-type, D109A, E182A, D288A, and Y328A variant enzymes were diluted to 0.8 mg/ml using Tris-HCl buffer (pH 8.5), and CD spectra were recorded for each protein in the far-UV (190–260 nm) and near-UV (240–310 nm) regions.

## Electronic supplementary material

Supplementary data
