# Supplementary material for: Structure-based prediction and identification of 4-epimerization activity of phosphate sugars in class II aldolases
Source: Sci Rep. 2017 May 16;7:1934. doi: 10.1038/s41598-017-02211-3 (PMC5434028; doi:10.1038/s41598-017-02211-3)
Supplement: Supplementary file 1 — Supplementary data [file 41598_2017_2211_MOESM1_ESM.doc]

**Supporting Information**

**Structure-based prediction and identification of 4-epimerization activity of phosphate sugars in class II aldolases**

Seon-Hwa Lee^1^, Seung-Hye Hong^1^, Jung-Ung An^1^, Kyoung-Rok Kim^1^, Dong-Eun Kim^1^, Lin-Woo Kang^2^ & Deok-Kun Oh^1,*^

^1^ Department of Bioscience and Biotechnology, Konkuk University, Seoul 05029, Republic of Korea.

^2^ Department of Biological Sciences, Konkuk University, Seoul 05029, Republic of Korea*.*

*Author for correspondence: deokkun@konkuk.ac.kr

**
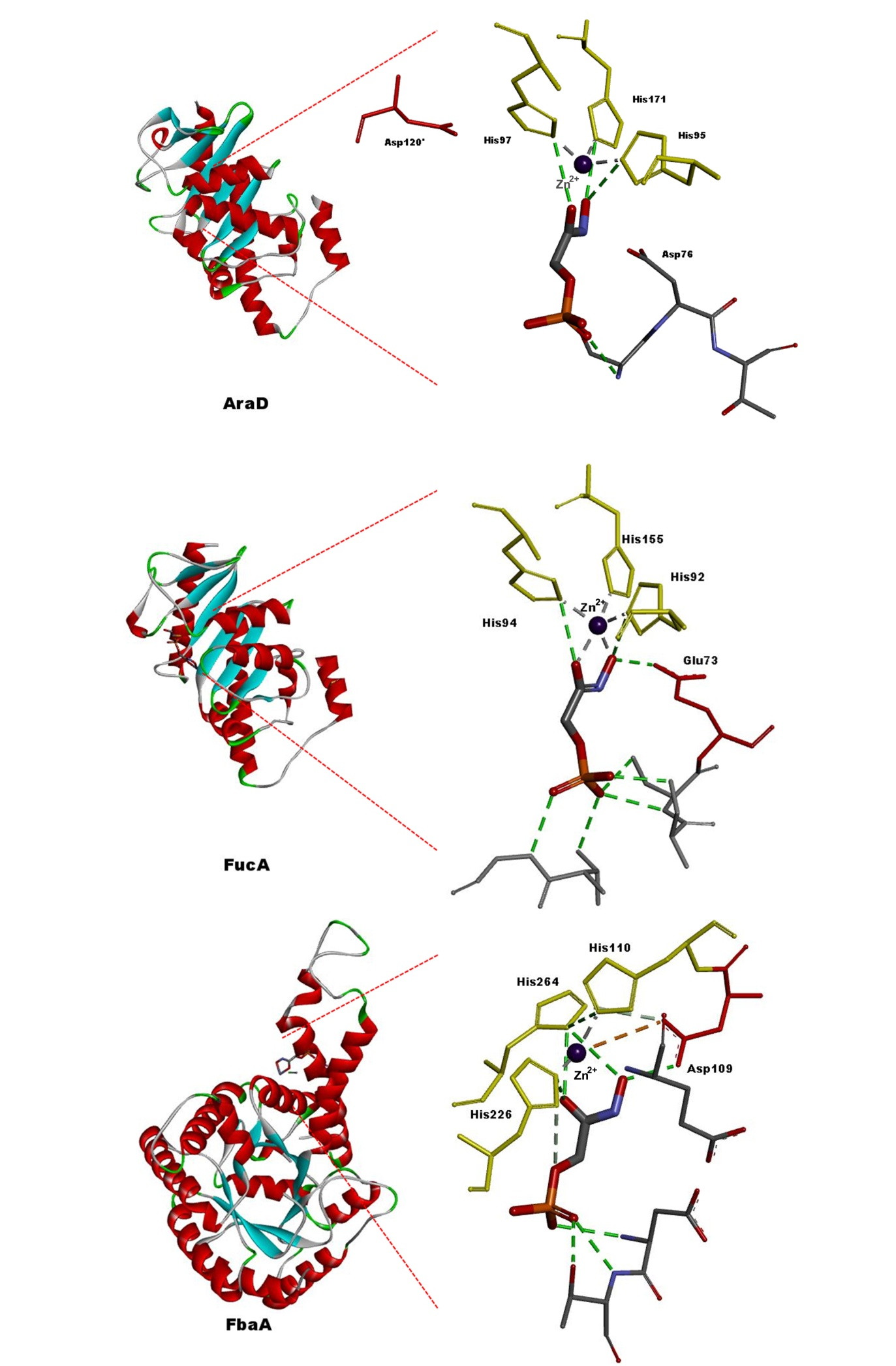

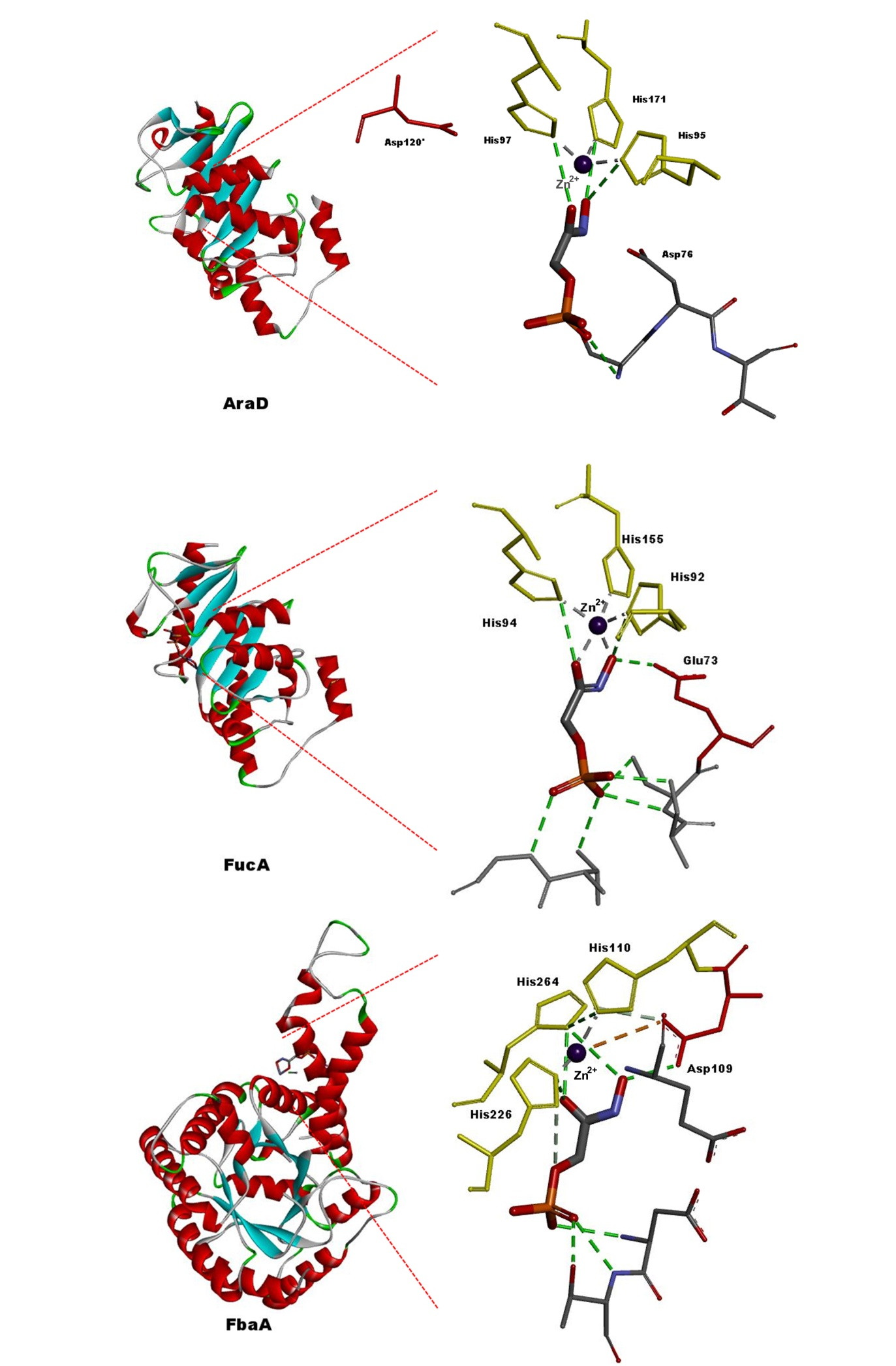
**

**A**

**B**

**C**

**
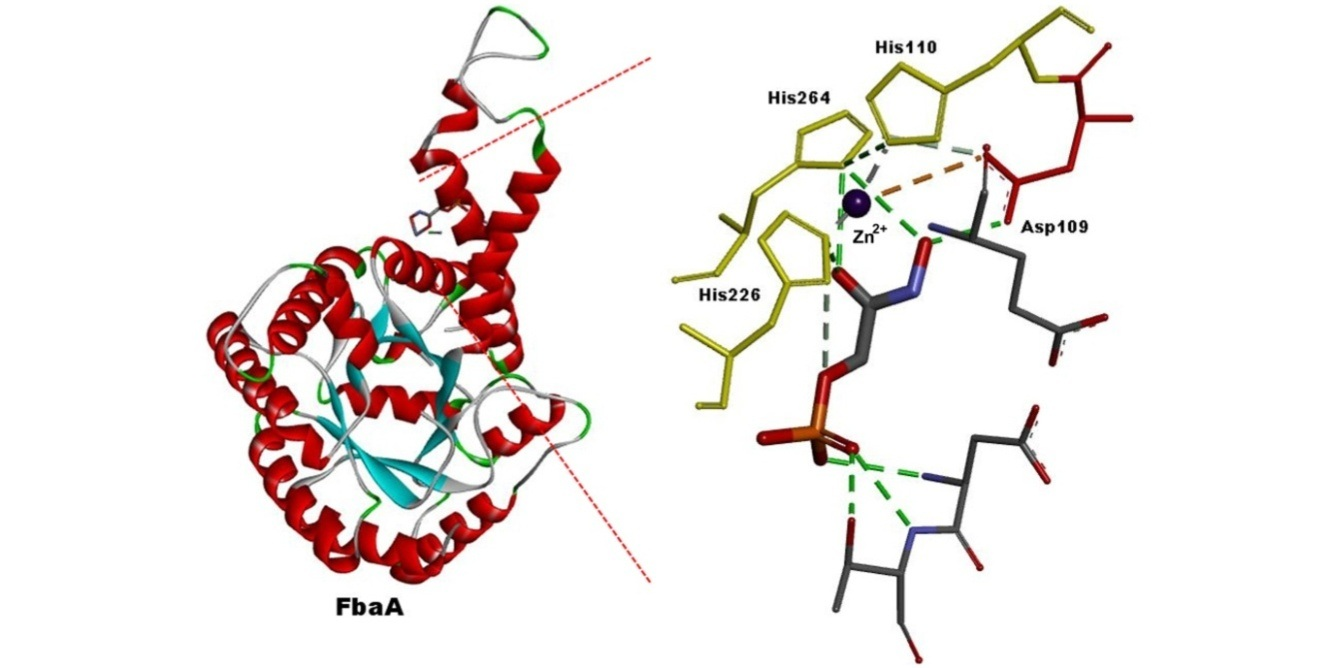
**

**D**

**
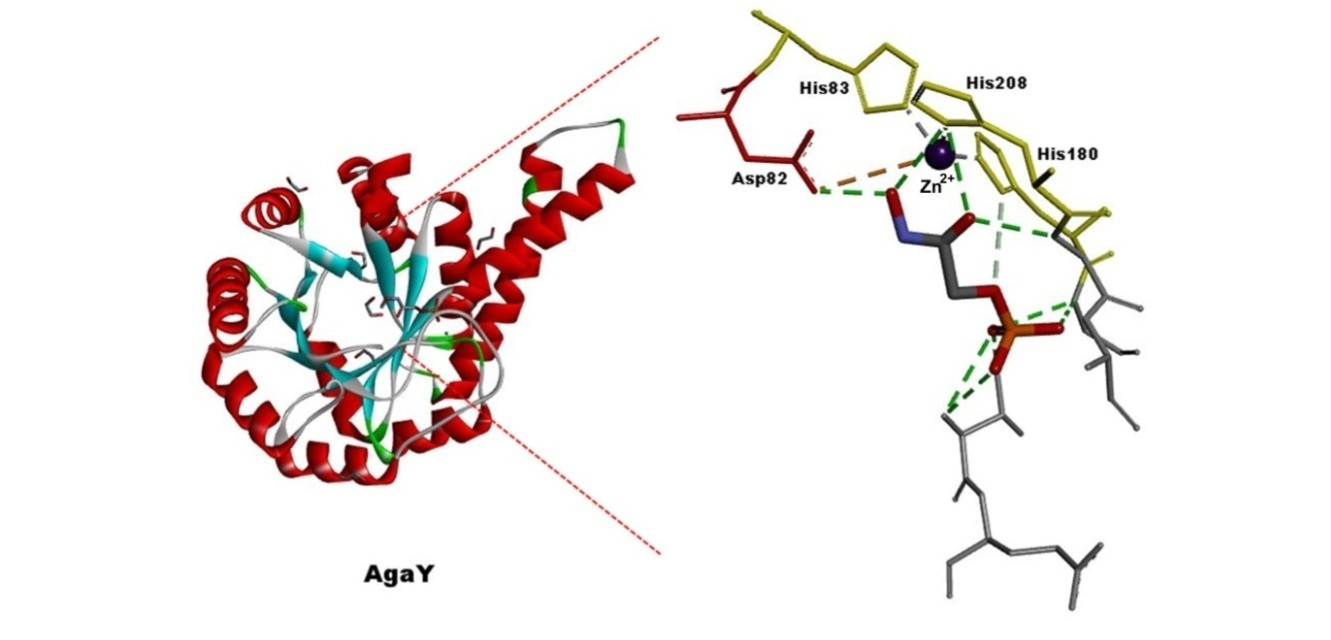
**

**E**

**Fig. S1. Structure of active sites in aldolases and epimerase. (A) AraD. (B) FucA. (C) RhaD. (D) FbaA. (E) AgaY.** Zn^2+^ in aldolases and epimerase is coordinated by a catalytic residue (Asp120 in AraD, Glu73 in FucA, Glu117 in RhaD, Asp109 in FbaA, and Asp82 in AgaY) and three histidine residues, which are spatially conserved in a tetrahedral arrangement. Green dashed lines represent hydrogen bonds. Oxygen, phosphorus, nitrogen, and carbon are shown in red, orange, blue, and grey, respectively. Zn^2+^, metal-binding residues, and catalytic residues are shown in purple, yellow, and red, respectively. Other amino acid residues are shown in light grey. The full names of AraD, FucA, RhaD, FbaA, and AgaY are given in Table S1.

**
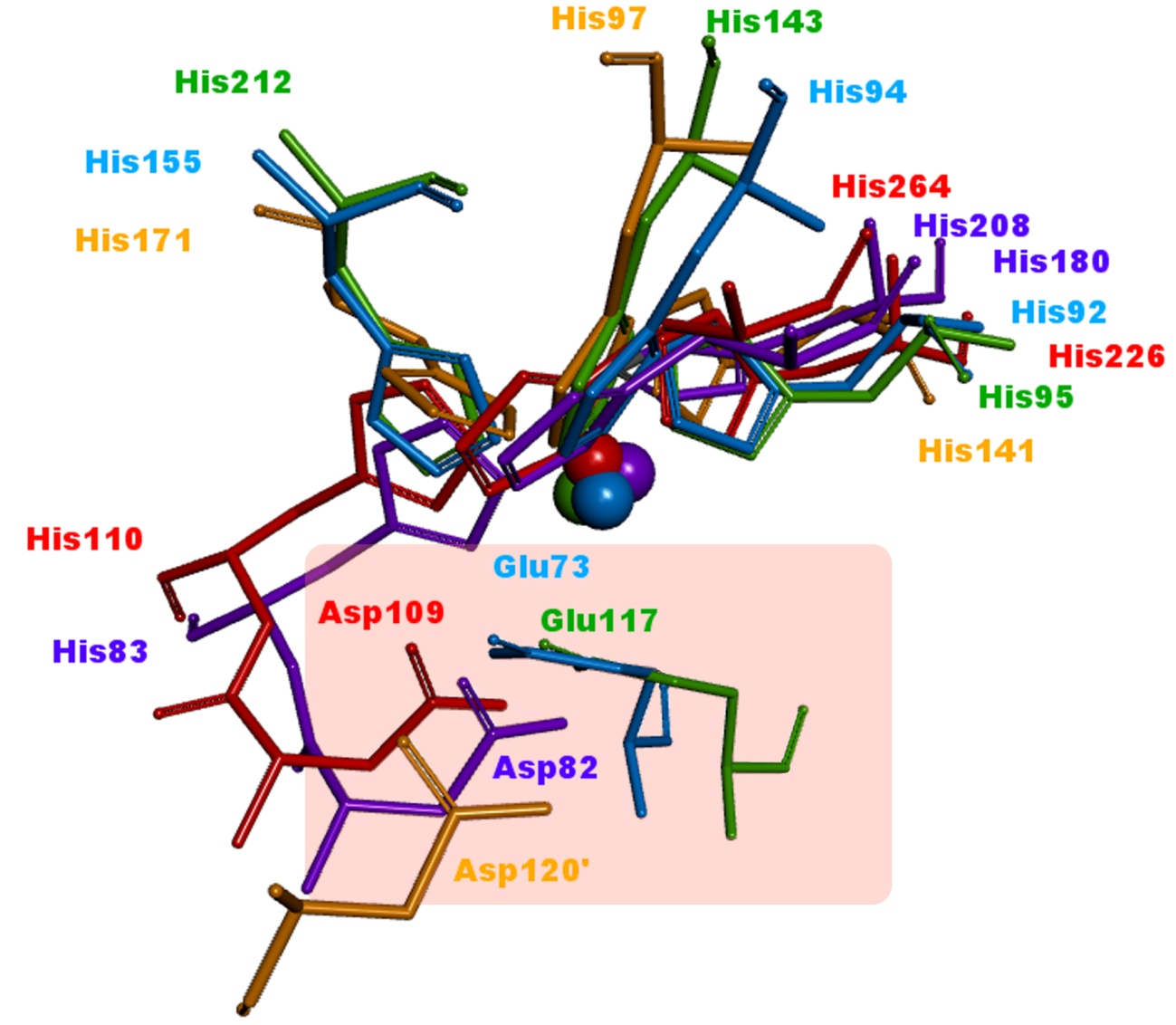
**

**Fig. S2. Superimposition of catalytic residues in AraD, FucA, RhaD, FbaA, and AgaY.** Structures are arranged based on the metal (Zn^2+^), which is coordinated by three histidines and a catalytic residue. The catalytic acidic amino acid in each enzyme was conserved as either glutamic acid or aspartic acid (pink box). The structures of AraD, FucA, RhaD, FbaA, and AgaY are represented in yellow, blue, green, red, and purple, respectively.

**
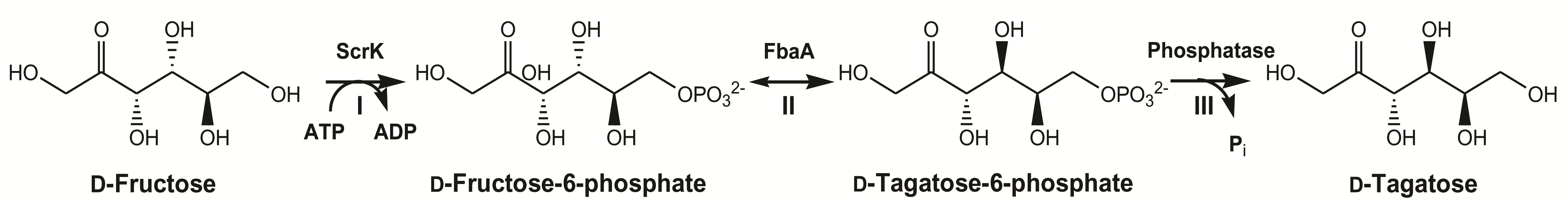
**

**Fig. S3. A three-step enzymatic cascade reaction for the production of tagatose from fructose.** Three enzymes, including ScrK (I), FbaA (II), and phosphatase (III), were involved in consecutive reactions from fructose to tagatose via F6P and T6P intermediates. (I) Fructose is phosphorylated to F6P in the presence of ATP by ScrK. (II) F6P is epimerized to T6P by FbaA with a yield of up to 90 %. (III) T6P is completely dephosphorylated to tagatose by phosphatase. Hexose phosphates and free hexoses were monitored by liquid chromatography. Phosphorylated hexoses F6P and T6P were eluted as described in the methods. Free hexoses were eluted at 30 °C using a mixture of 200 mM NaOH and water in a ratio of 5:95 (v/v) at a flow rate of 1 ml/min for 0–20 min.

**A**

**B**

**
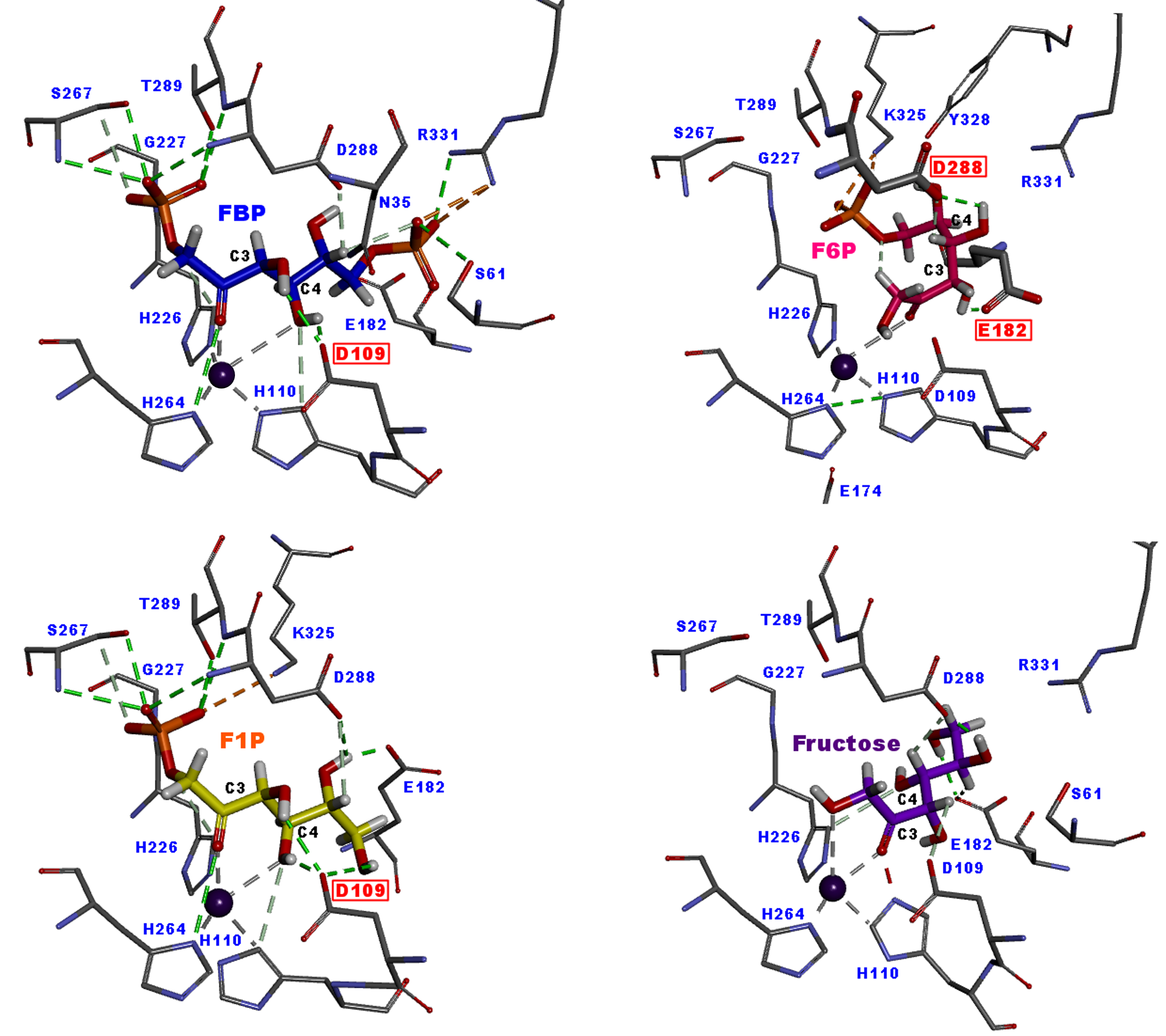
**

**C**

**D**

**Fig. S4. Structure of the FbaA active site complexed with a candidate substrate as determined** **by a computational docking model. (A) FBP (blue). (B) F6P (pink). (C) F1P (yellow). (D) Fructose (purple).** Green dashed lines represent interactions between the substrate and the catalytic residues (red).

**A**

**B**


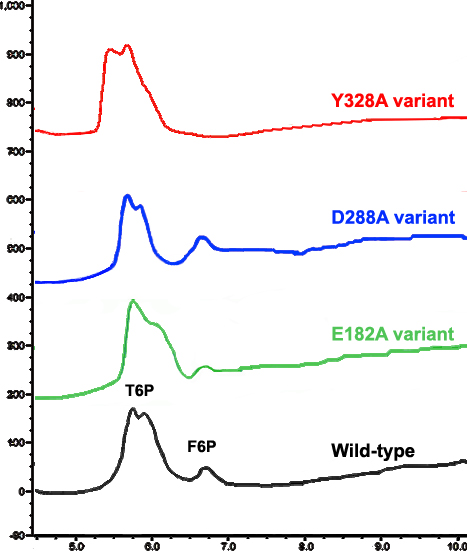

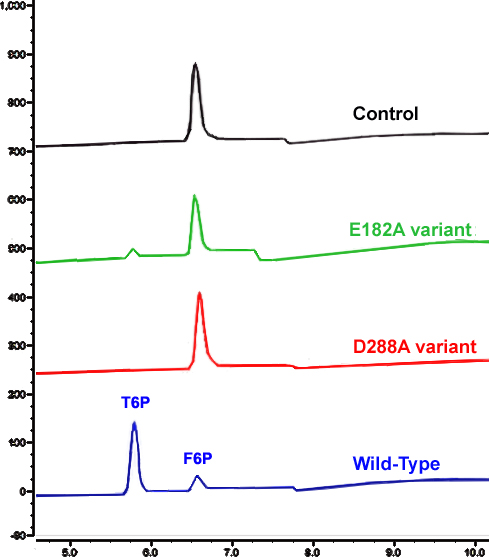


**Fig. S5. Determination of 4-epimerization of F6P and T6P by D288A and Y328A variants of *E. coli* FbaA, respectively.** The retention times for T6P and F6P in the Bio-LC were 5.7 and 6.8 min, respectively. **(A) Determination of F6P 4-epimerization.** The catalytic residue involved in the 4-epimerization of F6P to T6P was identified using the wild-type FbaA and its variants. Reactions were carried out at 50 °C in 50 mM Tris-HCl buffer (pH 8.5) containing 2 mM F6P and 1 U/ml wild-type FbaA (blue), E182A (green), or D288A (red) variants. F6P was used as the control. D288A showed no activity for F6P. **(B) Determination of T6P 4-epimerization.** The catalytic residue involved in the 4-epimerization of T6P to F6P was identified using the wild-type FbaA and its variants. Reactions were carried out at 50 °C in 50 mM Tris-HCl buffer (pH 8.5) containing 10 mM T6P and 20 U/ml wild-type FbaA (black), E182A (green), D288A (blue), or Y328A (red) variants. Y328A showed no activity toward T6P.


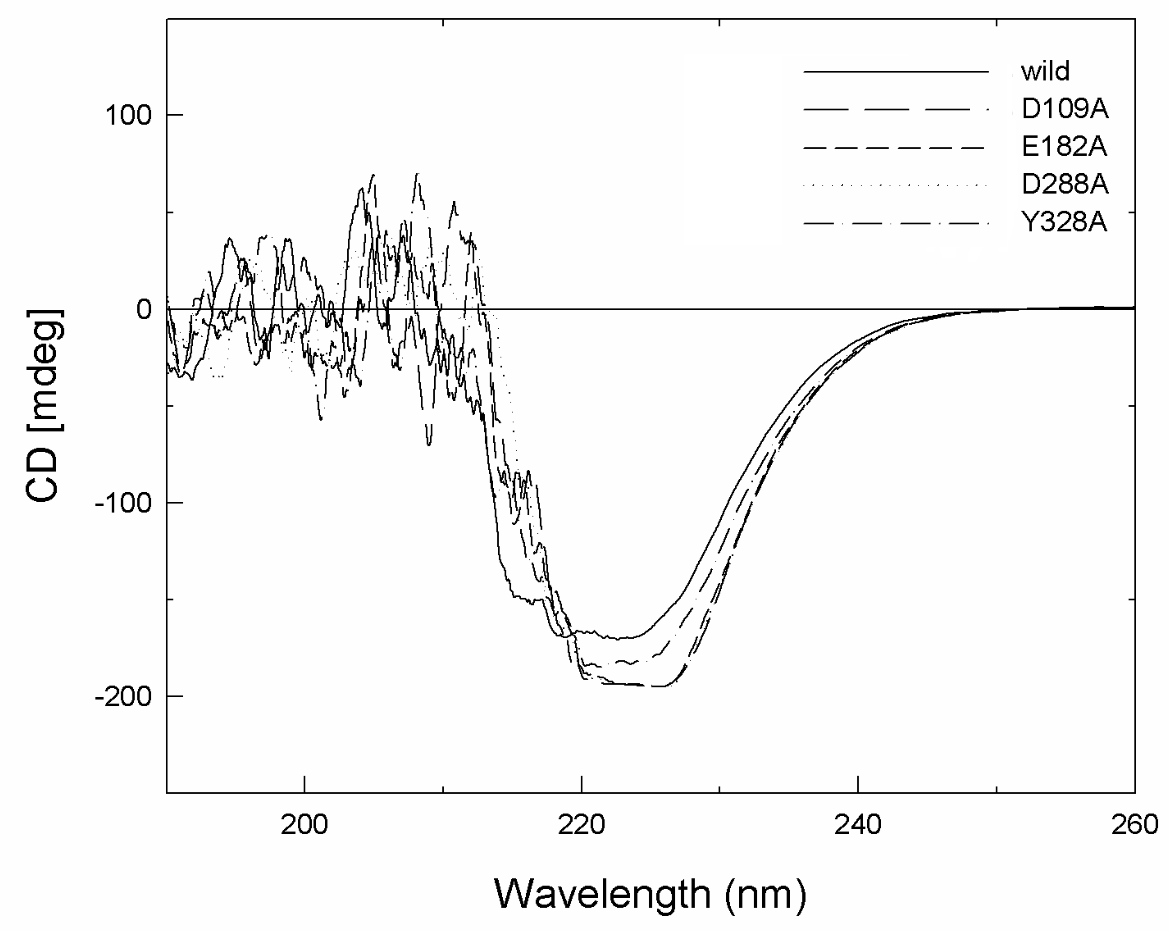


**Fig. S6. Near-UV circular dichroism spectra of the wild-type and variant FbaAs.**

**
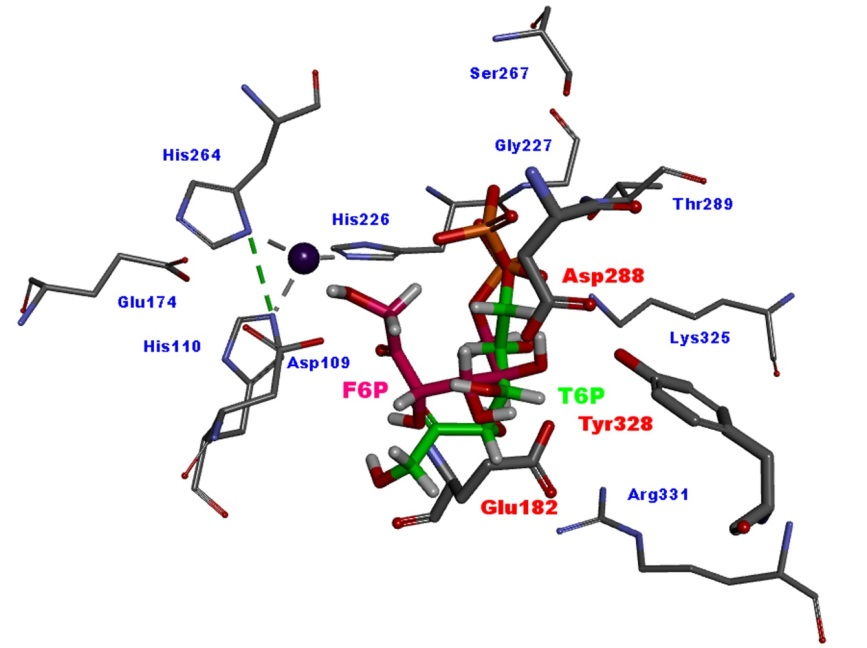
**

**A**

**B**

**
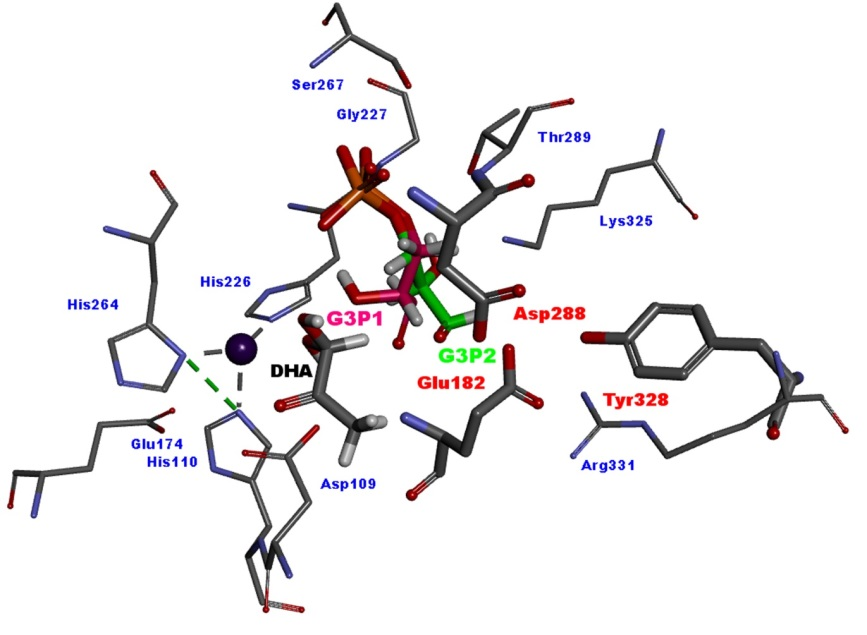
**

**Fig. S7. Docking of G3P, DHA, F6P, and T6P in the active site of FbaA.** The orientation of G3P pose 1 is similar to that of F6P, whereas the orientation of G3P pose 2 is similar to that of T6P. **(A) Docking of G3P and DHA in the active site of FbaA.** The orientations of G3P and DHA were similar to that of T6P, indicating that T6P is the preferred product. **(B) Docking of F6P and T6P in the active site of FbaA.** The two products, F6P and T6P, are generated from G3P poses 1 and 2, respectively.


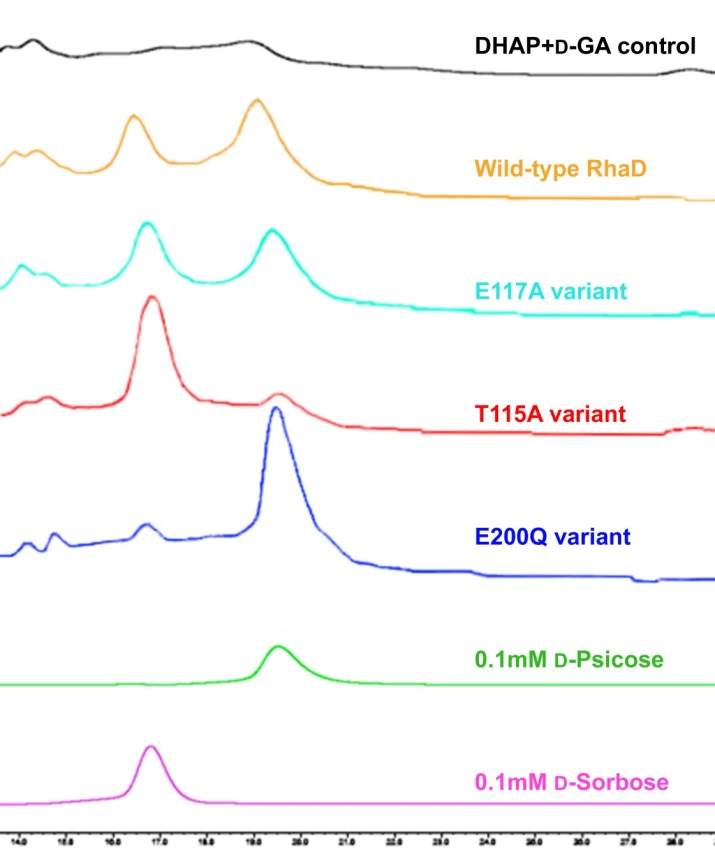


**Fig. S8. Synthesis of d-sorbose and d-psicose from DHAP and d-GA by the wild-type and variant RhaDs and phosphatase.** Bio-LC chromatograms show the reaction mixtures produced from DHAP and GA after the reactions of the wild-type RhaD and its variants and treatments of phosphatase. The retention times for d-sorbose and d-psicose were 16.7 and 19.5 min, respectively. The production pattern of E117A for d-sorbose and d-psicose was similar to that of the wild-type enzyme, indicating that Glu117 was not involved in 4-epimerization. d-Psicose synthesis from DHAP and GA by T115A significantly decreased whereas d-sorbose synthesis by E200A significantly decreased. Thus, Thr115 and Glu200 were newly identified catalytic residues with activities toward P1P and S1P for 4-epimerization, respectively. The reactions were carried in 50 mM Tris-HCl (pH 7.5) containing 10mM DHAP, 10 mM GA, and 1 mg/ml RhaD in the presence of 0.5 mM Zn^2+^ at 37 °C for 16 h. After condensation, the reaction mixtures were completely dephosphorylated. For the analysis of d-sorbose and d-psicose, the column was eluted at 30 °C with a mixture of 200 mM NaOH and water at a ratio of 8:92 (v/v) for 0–30 min, 50:50 (v/v) for 30–40 min, and 8:92 (v/v) for 40–45 min. Sorbose and psicose were detected at 16.7 and 19.5 min, respectively.

**
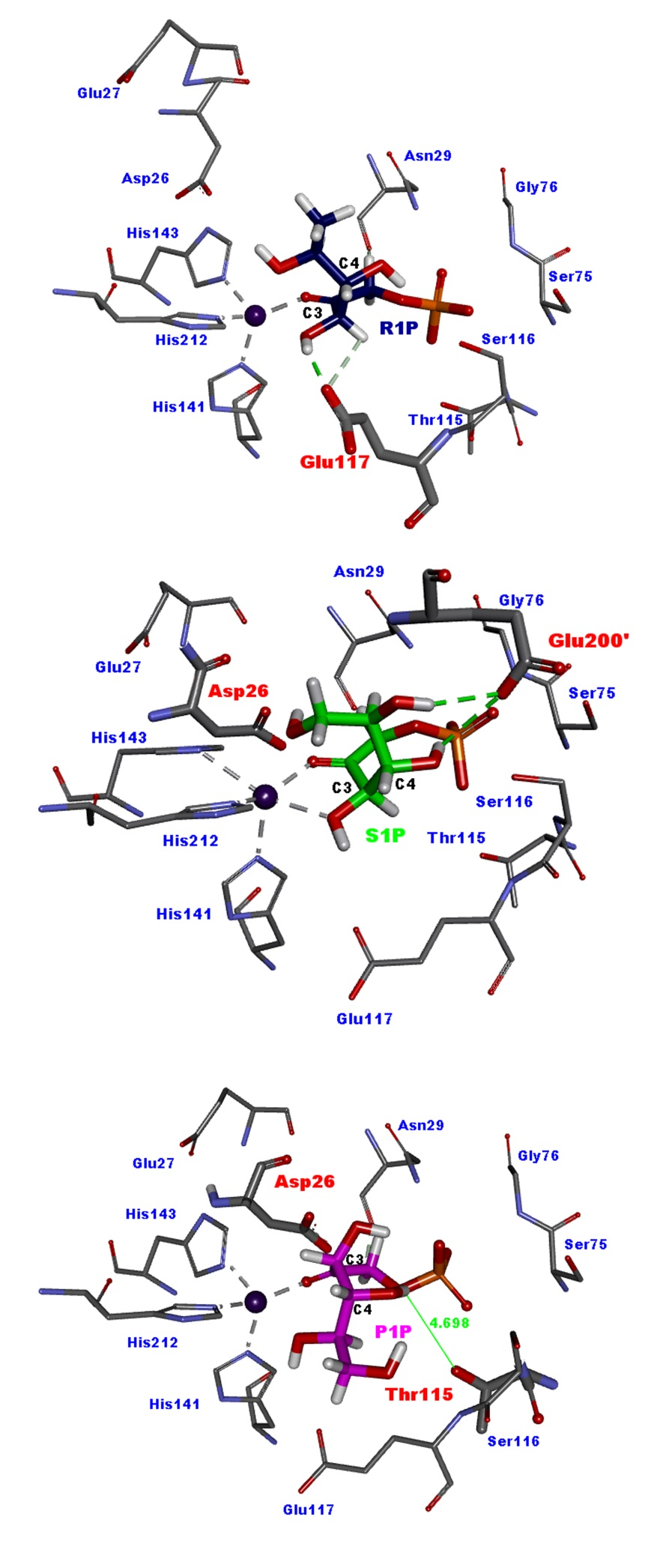
**

**A**

**B**

**C**

**Fig. S9. Docking of substrates in the active site of RhaD from *E. coli*. (A) Docking of l-rhamnulose-1-phosphate (R1P) in the active site of RhaD.** The catalytic residue of RhaD for aldol cleavage is Glu117. **(B) Docking of S1P in the active site of RhaD.** Glu200 forms a hydrogen bond with C4-OH of S1P. **(C) Docking of P1P in the active site of RhaD.** Thr115 was located close to the C4-OH of P1P within 4.69 Å. Green dashed lines represent the interaction between the substrate and catalytic residue. Grey dashed lines represent interactions with Zn^2+^. Metal-binding residues, Zn^2+^, and the catalytic residue are shown in blue, purple, and red, respectively.

**Table S1.** Sugar-related epimerases and aldolases in the SCOPe database.

| Functional classification | Protein name | Structural superfamily | | PDB code | Structural unit |
| --- | --- | --- | --- | --- | --- |
| Mono-, di-  phosphate  sugar-related | d-Ribulose-5-phosphate 3-epimerase | c.1.2.2: | d-Ribulose-5-phosphate 3-epimerase |  |  |
|  | l-Ribulose-5-phosphate 4-epimerase (AraD) | c.74.1.1: | AraD-like aldolase/epimerase, class II aldolase | 1JDI | 3-Layer sandwich |
|  | l-Fuculose-1-phosphate aldolase (FucA) | c.74.1.1: | AraD-like aldolase/epimerase, class II aldolase | 4FUA | 3-Layer sandwich |
|  | l-Rhamnulose-1-phosphate aldolase (RhaD) | c.74.1.1: | AraD-like aldolase/epimerase, class II aldolase | 1GT7 | 3-Layer sandwich |
|  | d-Fructose-1,6-bisphosphate aldolase (FbaA) | c.1.10.2: | Class II FBP aldolase | 1B57 | (β/α)8-Barrel |
|  | d-Tagatose-1,6-bisphosphate aldolase (AgaY) | c.1.10.2: | Class II FBP aldolase | 1GVF | (β/α)8-Barrel |
|  | Decameric fructose-6-phosphate aldolase/transaldolase | c.1.10.1: | Class I aldolase |  |  |
|  | Archaeal fructose-1,6-bisphosphate aldolase | c.1.10.1: | Class I aldolase |  |  |
|  | 2-Dehydro-3-deoxy-phosphogluconate aldolase | c.1.10.1: | Class I aldolase |  |  |
| NDP-sugar related | ADP-l-glycero-d-mannoheptose 6-epimerase | c.2.1.2: | Tyrosine-dependent oxidoreductases |  |  |
|  | GDP-mannose-3',5'-epimerase | c.2.1.2: | Tyrosine-dependent oxidoreductases |  |  |
|  | GDP-4-keto-6-deoxy-d-mannose epimerase/reductase | c.2.1.2: | Tyrosine-dependent oxidoreductases |  |  |
|  | UDP-*N*-acetylglucosamine 2-epimerase | c.87.1.3: | UDP-*N*-acetylglucosamine 2-epimerase |  |  |
|  | UDP-*N*-acetylglucosamine 4-epimerase | c.2.1.2: | Tyrosine-dependent oxidoreductases |  |  |
|  | UDP-galactose 4-epimerase | c.2.1.2: | Tyrosine-dependent oxidoreductases |  |  |
| Deoxy-sugar related | dTDP-4-dehydrorhamnose 3,5-epimerase | b.82.1.1: | dTDP-sugar isomerase |  |  |
|  | dTDP-4-keto-6-deoxy-glucose 5-epimerase | b.82.1.1: | dTDP-sugar isomerase |  |  |
|  | Deoxyribose-phosphate aldolase | c.1.10.1: | Class I aldolase |  |  |
|  | 2-Keto-3-deoxy gluconate aldolase | c.1.10.1: | Class I aldolase |  |  |
| Isomerase | Aldose 1-epimerase homologue | b.30.5.4: | Aldose 1-epimerase (mutarotase) |  |  |
|  | Putative *N*-acetylmannosamine-6-phosphate 2-epimerase | c.1.2.5: | NanE-like |  |  |
|  | *N*-Acyl-d-glucosamine 2-epimerase | a.102.1.3: | *N*-Acylglucosamine (NAG) epimerase |  |  |

**Table S2. Synthesis of hexose phosphates from DHAP or G3P as a donor and DHAP, DHA, or GA as an acceptor by FbaA.**

| Entry | Donor | Acceptor | Product | Concentration (mM) |
| --- | --- | --- | --- | --- |
| 1 | G3P | DHA | T6P  F6P | 0.16 |
|  |  |  |  | 0.08 |
| 2 | DHAP | G3P | FBP | 2.77 |
| 3 | DHAP | GA | F1P | 0.43 |

Reactions were carried out at 50 °C for 1 h in 50 mM Tris-HCl buffer (pH 7.0) containing 10 mM donor, 10 mM acceptor, and 20 U/ml FbaA.

**Table S3. Kinetic parameters of FbaA for FBP, F6P, and T6P.**

| Substrate | *K*_m_ (μM) | *k*_cat_ (min^−1^) | *k*_cat_/*K*_m_(mM^−1^ min^−1^) |
| --- | --- | --- | --- |
| FBP | 190±0 | 490±26 | 2,579±137 |
| F6P | 668±7 | 81±0.9 | 121±1.8 |
| T6P | 4,998 | 63.9 | 12.7 |

Data represent the means of three experiments and error bars represent standard deviation.

**Table S4. Equilibrium ratio of FbaA using seven different initial ratios of F6P and T6P with a total concentration of 10 mM.**

| Entry | Substrate (mM) | | Product (mM) | | Equilibrium ratio  (F6P:T6P) |
| --- | --- | --- | --- | --- | --- |
|  | F6P | T6P | F6P | T6P |  |
| 1 | 10.0 | 0.0 | 1.3 | 8.7 | 13:87 |
| 2 | 9.0 | 1.0 | 1.2 | 8.8 | 12:88 |
| 3 | 8.0 | 2.0 | 1.1 | 8.9 | 11:89 |
| 4 | 5.0 | 5.0 | 0.9 | 9.1 | 9:91 |
| 5 | 2.0 | 8.0 | 0.8 | 9.2 | 8:92 |
| 6 | 1.0 | 9.0 | 0.1 | 9.0 | 10:90 |
| 7 | 0.0 | 10.0 | 0.1 | 9.0 | 10:90 |
| Average |  |  |  |  | 10±2.5:90±2.5 |

Equilibrium ratios between F6P and T6P were determined by varying the ratio of initial substrates for FbaA. Reactions were carried out at 50°C for 24 h in 50 mM Tris-HCl buffer (pH 7.0) with 10 mM F6P and T6P and 20 U/ml FbaA. The data were obtained from one experiment.

| Entry | FbaA(U/ml) | PfkA(U/ml) | T6P (μM) | F6P(μM) | FBP(μM) |
| --- | --- | --- | --- | --- | --- |
| 1 | 0 | 0 | 0.0 | 10.0 | 0.0 |
| 2 | 0 | 5 | 0.0 | 2.0 | 8.0 |
| 3 | 0 | 10 | 0.0 | 0.7 | 9.3 |
| 4 | 0 | 30 | 0.0 | 0.5 | 9.5 |
| 5 | 10 | 0 | 4.5 | 5.5 | 0.0 |
| 6 | 10 | 5 | 0.3 | 2.5 | 7.2 |
| 7 | 10 | 10 | 0.0 | 2.2 | 7.8 |
| 8 | 10 | 30 | 0.0 | 1.5 | 8.5 |

**Table S4. Competitive enzymatic reactions between PfkA and FbaA for F6P.**

The distribution of T6P, F6P, and FBP was determined by varying the ratio of PfkA to FbaA. In entries 1–4, reactions were carried out in 50 mM Tris-HCl (pH 8.5) containing 10 μM F6P for 10 min at 65 °C with 0–30 U/ml PfkA. In entries 5–8, reactions were carried out under the same conditions with 10 U/ml FbaA and 0–30 U/ml PfkA. The data were obtained from one experiment.
